# A Hallowe’en Apparition

**Published:** 1903-10

**Authors:** 


### A HALLOWE'EN APPARITION.

FROM a recent copy of *Printer's Ink* it is learned with considerable surprise that the circulation of our esteemed contemporary, *The Georgia Journal of Medicine and Surgery,* published at Savannah, is given at over 4,000. This is a wonderful piece of information, and Georgia may well be proud of such thrift and enterprise in this department of her literature. Without endeavoring to controvert the statement upon which this rating was evidently based, we feel disposed to hazard a bunch of claims against old subscribers that there is some flaw in our colleague's arithmetic. As a copy of the Savannah publication has not been received at this office for six months, and that one three months overdue, preparations were being made to purchase a sprig of rosemary to drop upon its bier, under the merited assumption that Death's finger had touched it, or that it had been touched by the printers, amounting to the same thing. It is a relief to know that this assumption is unwarranted. Grief gives place to the milder feeling of regret at the irregularity with which the *Journal* is received. The poignancy of the regret is somewhat mitigated by the reflection that it is shared by three thousand nine hundred and ninety-nine other souls who are waiting, only waiting.

